# Managing Pharmaceutical Costs in Health Systems: A Review of Affordability, Accessibility and Sustainability Strategies

**DOI:** 10.3390/jmahp12040031

**Published:** 2024-12-10

**Authors:** Christos Ntais, Michael A. Talias, John Fanourgiakis, Nikolaos Kontodimopoulos

**Affiliations:** 1 Healthcare Management Program, School of Economics & Management, Open University of Cyprus, Nicosia 2220, Cyprus; christos.ntais@st.ouc.ac.cy; 2 Department of Management Science and Technology, Hellenic Mediterranean University, 72100 Agios Nikolaos, Greece; jfanourgiakis@hmu.gr; 3 Department of Economics and Sustainable Development, Harokopio University, 17676 Athens, Greece; nkontodi@hua.gr

**Keywords:** pharmaceutical expenditure, cost containment policies, drug price regulation, cost-sharing schemes, clawback, health technology assessment

## Abstract

Background: This paper reviews cost containment policies to control pharmaceutical expenditure either by regulating the pharmaceutical industry or targeting the demand side. Methods: The method used was the narrative literature review of studies which assessed the effect of pharmaceutical cost containment policies. Results: Governments worldwide have implemented a great variety of policy measures to manage pharmaceutical expenditure while ensuring fair access to essential medicines. Cost-sharing schemes, value-based pricing, reimbursement, reference pricing, payback mechanisms and the substitution of original drugs with generics and biosimilars are pivotal in these efforts, albeit with differing effectiveness across healthcare systems. Overall, it appears that any gains may be outweighed by the unfavorable effects of policies impacting patients. Although interventions have been created to improve physicians’ prescribing practice, they often achieve very minor benefits and at considerable cost. Policy measures pertaining to the regulation of the supply side must be supported by thorough evaluation in order to ascertain costs and effects and guarantee that unintended consequences are minimized. Conclusions: Policymakers frequently enact numerous laws and regulations to control pharmaceutical expenditure, even if there is limited evidence that they are cost-effective. The most crucial component of any policy’s success, regardless of the one selected, is its evaluation. Further research is needed to develop context-specific guidance that balances cost containment, equity and sustainability.

## 1. Introduction

The allocation of financial resources to healthcare systems remains a continuous challenge for governments and policymakers worldwide. Among the various components of healthcare spending, pharmaceutical expenditure wields significant influence over public and private healthcare budgets. Pharmaceutical costs have increased in real terms and as a proportion of overall expenditure in many healthcare systems in the developed world over recent decades [1]. To balance affordability, accessibility and sustainability, policymakers must traverse a complicated array of policies as healthcare systems struggle financially to fulfil the demand for medicines [2]. Governments have adopted a great variety of supply- and demand-side policies to control pharmaceutical expenses.

This study reviewed specific worldwide policies aimed at reducing drug-related spending and/or increasing the efficiency of pharmaceutical use by using resources best. We reviewed studies on the demand side of the pharmaceutical market that evaluate the impact of regulations meant to change prescribers’ and patients’ behavior. Regarding the supply side, we reviewed studies assessing the effect of laws restricting the pharmaceutical industry’s conduct, including authority over pricing and reimbursement.

## 2. Materials and Methods

The method used was the narrative literature review. The relevant literature was searched in the scientific databases PubMed and Scopus up to August 2024, using different combinations of the following terms: “pharmaceutical expenditure*”, “pharmaceutical expense *”, “pharmaceutical spend *”, “drug expenditure *”, “drug expense *”, “drug spend *”, “cost containment”, “cost constrain *”, “cost curtailment”, “cost reduction”, “cost sav *”, “cost control”, “cost cut *”. Articles published in peer-reviewed journals, written in English and including primary and/or secondary data, were considered for this review without any chronological and geographical restriction. Studies with no relevance to drug cost containment policies were excluded.

## 3. Results

### 3.1. Policy Measures to Control Pharmaceutical Expenditure

Policy measures to control pharmaceutical expenditure were classified into three categories: “Policies targeting the demand side: Patients”, “Policies targeting the demand side: Physicians” and “Policies regulating the supply side: pharmaceutical industry”. This classification was derived from the reviewed literature [3,4,5]. Table 1 lists several policy measures to control pharmaceutical expenditure. The most commonly used are described below.

#### 3.1.1. Policies Targeting the Demand Side: Patients

By limiting drug reimbursement and offering incentives to patients to cut back on their drug use, several interventions are utilized to lower the demand for pharmaceuticals.

- Cost-sharing schemes: Copayment

Patients who get a copayment must cover a portion of the difference between the amount they are reimbursed and the total cost of the medication. Copayments often consist of a set sum plus any discrepancy between the reference price and the actual price in countries where a set price is applied to a class of medications. Certain population groups, such as the elderly and those from underprivileged social backgrounds, can apply for special programs in various countries that provide full or increased reimbursement for essential prescription drugs.

Using panel data from European and non-European OECD member countries for the period 1990 to 2015, Berger et al. [6] evaluated the effectiveness of six types of demand-side expenditure control measures, such as physician-level behavior and system-level price control and substitution, alongside a proxy for cost-sharing. Their empirical analysis suggested that direct patient cost-sharing and some demand-side measures successfully slowed the growth of public pharmaceutical expenditure. Cost-sharing schemes emerged as an effective mechanism for slowing the growth of public pharmaceutical expenditure, but with a high risk of adverse effects.

Guindon et al. [7] examined the association of prescription drug insurance and cost-sharing with drug use, health service use and health. They found consistent evidence that having drug insurance and lower cost-sharing were associated with increased drug use, while a lack or loss of drug insurance and higher cost-sharing were associated with decreased drug use. Lee et al. [8] found that cost-sharing interventions such as user charges reduce pharmaceutical utilization and cut down on public expenditure by shifting costs to patients. On the other hand, by reducing the use of both essential and non-essential medicines and without adequate exemptions, such policies could disproportionately affect vulnerable groups.

Lee et al. [9] investigated how a new copayment plan in Korea affected the amount of money each patient spent on medications and the use of less expensive alternatives. They stated that with the implementation of a new copayment plan, the trend of rising drug expenditure per patient progressively declined. According to their calculation, after a year following the copayment increase, the amount spent on drugs per patient might drop by almost 12%. Furthermore, they found limited evidence of generic substitution brought on by the copayment hike. They concluded that patients’ access to costly therapies, regardless of clinical necessity, was being restricted by the new copayment structure, rather than saving money through the anticipated mechanisms, such as generic substitution. This sparked concerns about possible harm to public health as well as a loss of equality in the use of medications.

- Withdrawing from reimbursement (Delisting)

Reducing pharmaceutical spending may also involve taking a medicine off of reimbursement. Removing payments for medications that do not fulfill safety or efficacy standards does not always result in better medication usage overall. Sumerai et al. [10] examined the impact of the government’s decision to stop funding 12 classes of medications with dubious effectiveness. The study involved four cohorts of regular users of these drugs as well as a random sample of the Medicaid population (*n* = 390,465). During the 42-month trial, prescription rates increased from 0.86 to 1.00 monthly prescriptions per enrollee; this is despite the fact that withdrawn medications constituted 7% of prescriptions in the base year. There was also no discernible decrease in total drug use or expenditures following the rule. After accounting for prior patterns, there was an estimated rise of 33.7 prescriptions for substitute pharmaceuticals for every 1000 participants per month, which more than compensated the expected decrease of 21.7 prescriptions for study drugs. There were both non-desirable and desirable therapeutic substitutes. The authors concluded that used alone, curtailment of reimbursement for marginally effective therapies results in both desirable and unintended clinical substitutions and may not reduce costs.

#### 3.1.2. Policies Targeting the Demand Side: Physicians

Several initiatives have been introduced to improve the efficiency of physicians’ prescribing behavior. These include providing information and feedback to physicians on prescription analysis and costs, budget constraints, prescribing guidelines and incentive systems.

- Information and feedback to physicians

Many national health funds disseminate information on prescribing behavior to physicians in order to increase their awareness of costs. Comparisons are made between practices and the local average, and written warnings and/or penalties are issued if a practice’s costs are significantly higher than the local average for prescribing by colleagues. However, in many countries, this information may be ignored. This is the case in Greece, where data on prescription costs in relation to consultations are tracked and fed back to physicians to enable them to monitor their own prescribing patterns [11,12,13]. All of these systems are advisory and provide information on prescription volume and cost, but they do not provide information on the cost-effectiveness of prescribing and may therefore penalize the use of expensive drugs that may have benefits worth the extra cost.

- Budgetary restrictions—Incentive systems

Budget initiatives have suggested that drug costs can be managed through financial incentives or penalties for physicians working within a global drug budget. In some countries, fixed budgets for primary care physicians provide an explicit incentive to contain costs, which encourages the prescription of generic medicines [14]. Incentives may be designed to reward physicians who spend less, penalize physicians who spend too much or both. In some US health maintenance organizations, physicians have discretionary salary increases that they receive if they have used healthcare resources efficiently.

- Prescription guidelines

Antibiotic and non-steroidal anti-inflammatory drug prescriptions have significantly decreased since the implementation of prescribing guidelines for physicians on diagnosis and treatment [15]. Guidelines are employed as a tool for budgetary control in some countries since they are designed such that the total number of prescriptions does not exceed the budget. It should be underlined that prescribing guidelines become nothing more than worthless voluntary guidance schemes in the absence of incentives to follow them, such as awards for physicians who follow them and sanctions for those who noticeably violate them. There are circumstances in which the costs of implementing prescription guidelines may surpass the savings that result from lower prescribing. This was discovered in a study that examined prescribing guidelines in general practice [16].

#### 3.1.3. Policies Regulating the Supply Side: Pharmaceutical Industry

- Pharmacoeconomic evaluation/Value-based pricing/Health Technology Assessment

In order to assess the value of pharmaceuticals—which is typically measured in terms of outcomes (such as quality-adjusted life years, QALYs)—pharmacoeconomic studies frequently include a cost-effectiveness analysis. Moreover, cost–benefit analysis and cost minimization are forms of pharmacoeconomic evaluation. Pharmacoeconomic evaluation is a very helpful method for determining a medicine’s worth for the money.

Tisdale et al. [17] assessed the availability and quality of cost-effectiveness studies for 250 prescription drugs with the greatest Medicare Part D spending in US-based adult patient populations. Public spending on these 250 drugs totaled US$122.8 billion in 2016 (84.1% of total spending). There were 280 unique cost-effectiveness analyses for these drugs, representing data on 135 (54%) of the 250 drugs included and 67% of public spending on the top 250 drugs. The 115 drugs (46%) without cost-effectiveness studies accounted for 33% of public spending on the top 250 drugs. Of the 280 available studies, 128 (45.7%) were industry-sponsored. Surprisingly, most studies (250 [89.3%]) did not meet the minimum quality requirements. In conclusion, a substantial proportion of 2016 Medicare Part D spending was for drugs with absent or low-quality cost-effectiveness analyses.

Value-based pricing and outcome-based agreements set drug prices according to health outcomes and their potential economic consequences. Value-based pharmaceutical contracts (VBPCs) are performance-based reimbursement agreements between healthcare payers and pharmaceutical manufacturers in which the price, amount or nature of reimbursement is tied to value-based outcomes. VBPCs are often complex, and the nature of who benefits and in what ways can be unclear. Kannarkat et al. [18] recommended taking a patient-centered approach when developing VBPCs and tying VBPCs to more overarching payer drug cost reduction strategies.

More and more countries are considering the economic parameter when determining whether to reimburse drugs. Health Technology Assessment (HTA) is a multidisciplinary approach that provides a systematic, transparent, unbiased and robust summary of information regarding the medical, social, economic and ethical issues associated with using health technology. HTA is a crucial instrument for evaluating pharmaceuticals’ clinical efficacy and cost-effectiveness. These evaluations impact reimbursement choices, directing healthcare spending toward medications that offer good value for the money. For example, using HTA to reveal the added value of pharmaceutical treatments in personalized medicine is challenging, which in turn may well impact reimbursement processes [19].

- Reimbursement—positive lists for reimbursed medicines

Many governments use lists of pharmaceuticals categorized by their reimbursement status to limit the number of drugs that are publicly reimbursed [20,21]. These can contain pharmaceuticals that are specifically prohibited from reimbursement (negative list) or those that are eligible for reimbursement (positive list). Lists of reimbursement may be directed toward the inpatient sector (often referred to as hospital pharmaceutical formulary) or the outpatient sector (generally, positive or negative lists) or both. Reimbursement status decisions are made on the basis of safety and effectiveness, professional judgment and, occasionally, cost-effectiveness. A medication is delisted when it is removed from a reimbursement list (positive) and thus becomes not eligible for reimbursement.

Internal reference pricing, i.e., the process of determining or negotiating reference drug prices by comparing them to the costs of therapeutically equal or identical medications sold in the country, is a reimbursement policy used in several countries worldwide [22,23]. In reference pricing systems, patients pay any discrepancy between the actual retail price of the drug prescribed and the reference price, in addition to any copayments. A reference price is defined for identical medicines (ATC level) that are clustered (reference group). There are two types of reference groups: ATC 4- by therapeutic use and ATC-5 by active ingredient. Where the reference price is based on the lowest priced drug(s) in the group, reference pricing appears to be one of the few strategies likely to be effective at encouraging physicians to use the least expensive agents as first-line therapy and utilize more expensive agents in those who experience side effects or poor efficacy [24]. In addition, when a reference price scheme is introduced, certain manufacturers may decide to lower the price of their pharmaceuticals that are priced higher than the reference price because they believe that patients will not pay the higher cost. On the contrary, some may charge more than the reference price and depend on the willingness of the patients to cover a portion of the cost. Studies on reference pricing have provided evidence that this could lead to cost reductions [8]. However, these are not attained by industry cutting or restricting pricing, nor by a decrease in the total number of prescriptions issued; instead, they are the result of specific usage changes and cost shifting to patients, which has a negative impact on the equality of access to medications [8]

- Price regulation

External reference pricing, sometimes referred to as international reference pricing, is a method of price regulation in which a government looks at the price of a drug in other countries to establish a standard for determining or negotiating the cost of a pharmaceutical product in its own country [22]. Typically, a list of countries with GDP per capita comparable to purchasing power parity or other predetermined standards is used to select reference countries. The chosen countries are used as a reference for both the setting of new prices and the periodic price review, which involves evaluating the price, usually in relation to the prices of the same medications in other countries, to take into account developments like the introduction of new medications into the market and price adjustments in other countries.

In 2013, the Chinese government set the maximum retail prices for antibiotics included in the national reimbursement list. Wushouer et al. [25] found that the average daily cost of the price-regulated group decreased significantly following government price regulation in comparison to antibiotics with no price restriction. A favorable trend shift was observed in the average hospital spending on price-unregulated antibiotics, while the average hospital monthly spending on price-regulated medicines also declined rapidly.

- Budget programs and periodic price adjustments

Lin et al. [26] assessed how a global budget program and drug pricing adjustments to Taiwan’s National Health Insurance scheme affected spending and antibiotic prescription trends. Antibiotic costs rose by 54.1% throughout the first five years of the global budget program despite a fall in volume of 11.2–12.3%. The main factor driving cost growth was therapeutic choice. The factor influencing treatment choice steadily declined in the second and third 5-year periods, whereas the cost of antibiotics was constant despite a modest rise in the number of prescriptions issued. In contrast, periodic price adjustments steadily reduced costs by 21.9 to 39.9% without appreciably increasing the volume of drugs.

Based on a literature review, Mills and Kanavos [27] found that fixed global budgets for total pharmaceutical expenditures are primarily utilized to promote cost-containment. Nevertheless, their use frequently limits flexibility in allocating the total health budget. Furthermore, since disease-specific budgets are routinely exceeded, it is unlikely that they will encourage fiscal sustainability. In contrast, product-specific and prescribing budgets can significantly enhance microeconomic efficiency, though the evidence supporting their benefits is conflicting.

- Tendering, centralized drug procurement programs and national drug price negotiation policies

Governments ask manufacturers to submit quotes for a specific contract through a procedure known as tendering, which often involves a competitive bidding process. It is frequently used to lower the cost of medications that already face competition in the market. It typically occurs toward the end of the pricing process, after the government has established a baseline price to reference the bids. It usually involves some negotiation and functions concurrently as a procurement strategy to support supplier and volume decisions for certain medications.

A pilot program for centralized drug procurement was launched in China in September 2019 to lower medicine costs through volume-based procurement and the substitution of branded drugs with approved generic ones [28]. The volume of pharmaceuticals that won bids surged dramatically once the policy was put into place, while the volume of branded and generic drugs soon fell. The defined daily drug cost of medications sold under the same generic name dropped dramatically (by about 25% in the case of products that won the bid). Overall, the strategy showed promise regarding cost savings and price reductions, and it hastened the shift from branded to generic medications [29]. Additionally, following policy intervention, the general quality level of drug usage among the Chinese population increased, particularly in primary healthcare settings [30]. Yuan et al. [31] examined how the national volume-based procurement program affected the costs of specific antiviral medications in China and found that it successfully lowered prices and overall spending.

China introduced a national drug price negotiation policy in 2018, adding 17 cutting-edge anticancer medications to the national reimbursement drug list. From October 2017 to December 2019, Cai et al. [32] looked at changes in these medications’ availability, utilization, defined daily dose cost and affordability using monthly pharmaceutical procurement data from 1039 hospitals. Following the implementation of the policy, their availability grew by 1.23% every month and by 7.88% immediately. These medications’ defined daily dosage cost dropped by US$109.09 immediately. It stayed consistent subsequently, but the utilization of these medications increased by 11.44 defined daily doses immediately and by 3.54 defined daily doses each month after the policy’s implementation.

Main et al. [33] examined the policy processes underlying New Zealand’s exceptional track record of cost savings in public pharmaceutical spending. They found that a group of managers at the county’s drug reimbursement agency, the Pharmaceutical Management Agency, and the Health Technology Assessment Agency—which negotiated pharmaceutical prices on behalf of the public payer—dominated decision making over pricing and reimbursement policies. These managers used various pricing techniques in formal negotiations over patented pharmaceutical products. The most well-noted ones were “bundling”, which consists of discounted package deals for several pharmaceuticals, and “play-off tenders”, in which two or more pharmaceutical companies bid for exclusive contracts. By contrast, “blind tenders”, which are an annual bidding procedure for supply contracts, represented the primary pricing approach for generic medications.

- Special pricing agreements

Governments and manufacturers enter into legal contracts known as special pricing agreements, which offer an opportunity for payers and pharmaceutical companies to align on value, optimize speed to patients and share risk [34].

- Budget cap and payback schemes (Clawback)

The amount that pharmaceutical companies must reimburse the state when public spending on medications is above the allocated funds is known as clawback. Mitkova et al. [35] examined the impact of a budget capping policy on Bulgaria’s total pharmaceutical expenditure. In their opinion, a budget cap was a reasonably effective tool; nonetheless, the high degree of overspending and the payback amount indicate that the market environmental risk elements were not well foreseen or implemented.

The Greek government implemented a clawback system 2012 to regulate public pharmaceutical outpatient spending. Since 2016, Greece has also embraced the clawback as a policy to limit pharmaceutical spending at National Health System hospitals. Letsios et al. [36] examined the effects of this strategy on public hospital operation, the continuous supply and prudent use of medications and the financial viability of these facilities. They concluded that hospitals in the Greek National Health System are under constant pressure to spend excessive amounts on pharmaceuticals, and this, combined with their tight budgets, puts both the pharmaceutical industry’s survival and the uninterrupted supply of medications to public hospitals at risk.

- Escalating obligatory discounts (rebate)

Rebates, which are a form of escalating obligatory discounts to public hospitals and national healthcare funds based on volume, commonly involve the government and patient paying the list price at the point of purchase, with the rebate returning to the government’s general revenue as a payback within a specified time frame. The cost to patients when price reductions are carried out through rebates (rather than from a transparent decrease in the list price) could be higher than the drug’s actual cost [37].

- Generics pricing

It is standard practice to set the price of a generic drug in proportion to the original medication, typically at a percentage lower than the original medicine price (price link). This generic price link policy may have different designs, with varying percentages for the various generics (first generic to hit the market, second generic, etc.) [38]. In certain situations, the policy may also require the prices of original medications to drop.

- Prescription generic drug price reduction

The Canadian government introduced a generic pricing strategy in 2013 to rein in growing pharmaceutical costs. The program resulted in a nearly 50% price reduction for certain prescription generic medications. Li [39] examined how this Canadian pricing policy affected senior drug use and drug expenditure. The generic price reduction had a positive impact on lowering per-capita prescription costs. The interplay between demand-side incentives and restrictions was critical to the outcome. Similarly, Kim et al. [40] found that Korea’s monthly drug spending immediately decreased due to large generic price reductions and incentives for more effective drug prescriptions.

- Substitution of original drugs with generics and biosimilars

It has been established that using generic medications can help contain the rising costs of prescription drugs [41]. Most countries promote generic medications, and some even let pharmacists use generic drugs instead of branded drugs.

To illustrate the financial effects of the launch of a biosimilar trastuzumab in the treatment of human epidermal growth factor receptor 2-positive breast cancer, Chai et al. [42] built a budget impact model from a payer’s perspective in China. According to their study, costs would go down if a trastuzumab biosimilar was adopted. Over five years, the predicted total cost reductions ranged from US$10.3 million in the first year to US$60.8 million in the fifth, with an average of US$46.6 million. An additional 654–3858 breast cancer patients may benefit from these cost savings.

Gomes et al. [43] estimated the financial effects of implementing reimbursement regulations in Ontario, Canada, requiring the replacement of innovator biologics indicated for inflammatory diseases with less expensive biosimilars. According to their projections, from 2018 to 2020, a forced substitution of biosimilars might have potentially impacted 7209 patients and resulted in savings of US$238.6 million. Additionally, if an adalimumab biosimilar were to become available, 12,928 people would be subject to a forced substitute, resulting in a US$34.2 million savings over a three-year period. In addition, a new-user substitution would have affected 757 patients and saved US$645.9 million.

- Policies regulating mark-ups in the pharmaceutical supply and distribution chain

Drug wholesalers and retailers are subject to profit margins preset by most governments. Limiting markups across the entire pharmaceutical supply and distribution chain could be a valuable strategy for managing drug costs and achieving more widespread access to medications. Joosse et al. [44] concluded that mark-up regulation laws might help bring down the cost of pharmaceuticals while a critical component of mark-up laws’ potential effectiveness was how they were designed.

- Policies promoting drug price transparency

It has been claimed that policies encouraging drug pricing transparency are a crucial strategy for reducing the cost of medications and improving access to them. Joosse et al. [45] investigated the efficacy of pricing transparency policies in controlling pharmaceutical product costs. They found that while there was conflicting evidence about the effectiveness of a cost-feedback approach to prescribers, it may be possible to reduce drug prices in the near term through public disclosure, with potential long-term advantages.

## 4. Discussion

In many developed healthcare systems, spending on pharmaceuticals has climbed both in absolute terms and as a percentage of total spending during the past few decades. Governments have implemented a wide range of supply-side and demand-side measures to keep pharmaceutical costs under control.

Overall, data regarding the impact of user charges show that cost-sharing lowers payer spending on prescription drugs; however, this is accomplished by decreasing drug usage and passing along costs to patients. User charges can also reduce the use of essential pharmaceuticals, even though the decrease in the use of less essential drugs is higher. It should be no surprise that the utilization reduction becomes higher with the cost-share. According to Gibson et al. [46], individuals receiving a new diagnosis had a higher likelihood of quitting their medication. The existence of safety net exemptions determines how user charges affect vulnerable groups, such as the elderly, the seriously ill and those with low income [47]. Policies that target patients—especially those involving user charges—may have unfavorable effects that outweigh their advantages.

Evaluating physician-related policies such as incentive programs, budgetary constraints and prescription guidelines, Lee et al. [8] found that the evidence supporting these policies is of varying quality. Although some studies have shown unexpected outcomes, such regulations can reduce pharmaceutical costs and change usage between categories, increasing the use of generics. The authors concluded that, while a variety of strategies may somewhat influence prescription, it is crucial that solid data on efficacy and cost-effectiveness support communications to prescribers.

According to O’Malley et al. [48], rewards for generic prescribing in the USA did not affect prescribing, while according to Martens et al. [49], Dutch physicians who followed prescription guidelines received bonuses that had little impact on prescribing practices. In general, it has been shown that prescriber incentives result in only minor benefits, occasionally at significant expense.

The rising cost of pharmaceuticals is associated with physicians prescribing expensive medicines [50]. Governments must put in place a mechanism that allows physicians and hospitals to save money in a way that is both effective and efficient, including by prescribing comparable medications at a reduced cost. More attractive incentive programs would probably influence physicians to prescribe in a way that is more cost-sensitive. A visit from the appropriate authorities to discuss prescription rates should be given to physicians whose prescription level exceeds a preset ceiling, e.g., a specific rate above the average.

Price control measures are typical approaches to cost containment in regulating the pharmaceutical industry; successful cost containment necessitates management of both pharmaceutical pricing and the volume of prescription medication. Price limitations could not always encourage the efficient use of medications unless they are coupled with closely monitored economic evaluation. It should be mandatory for pharmaceutical companies to submit applications for reimbursement with a financial assessment attached. New medications should be sold for the same price or less if they do not clearly outperform currently available ones. If clinical trials demonstrate superiority, incremental cost-effectiveness should be evaluated to ascertain whether a product is cost-effective at the desired price. Guidelines should also be published for the economic analyses that must be included in submissions for reimbursement listing.

As a narrative review, this study is inherently susceptible to selection bias. To minimize selection bias, we conducted a comprehensive literature search across the PubMed and Scopus databases using predefined search terms. The inclusion of studies was guided by relevance to the objectives, and all four authors critically reviewed interpretations to ensure balanced perspectives. We aimed to broadly represent the literature by including studies from different health system contexts and perspectives, encompassing both high-income and low- and middle-income countries. Future research could consist of systematic reviews or meta-analyses to provide a more comprehensive and quantitative synthesis of evidence.

## 5. Conclusions

After decades of high rises in pharmaceutical spending, cost-containment measures have been implemented, such as limiting pharmaceutical demand and controlling their supply through governmental initiatives. Overall, any gains may be outweighed by the unfavorable effects of policies impacting patients, especially cost-sharing schemes. Although interventions have been created and assessed to improve prescribing practice, they often achieve minor and costly benefits. Regulations about the supply side of the pharmaceutical market, i.e., the pharmaceutical industry, must be supported by thorough evaluation to ascertain costs and effects, guarantee that unintended consequences are minimized and create the body of evidence needed to support related policies.

Policymakers frequently enact numerous laws and regulations to limit spending on pharmaceuticals, even if there is limited evidence that they are cost-effective. Reimbursement in both public and private healthcare systems has to be based on data demonstrating the cost-effectiveness of medicines, and utilization needs to be limited by regulations governing the conduct of physicians, patients and the pharmaceutical industry. Societal value merits consideration when determining whether and how to make medicine affordable and accessible to patients; a drug worth its price to society should not be rendered inaccessible to patients by imposing high out-of-pocket costs or restricting coverage based on narrow assessments. The most crucial component of any policy’s success, regardless of the one selected, is its rigorous evaluation.

## Figures and Tables

**Table 1:** Policy measures for the cost containment of pharmaceutical expenditure.

***Policies Targeting Patients***	Copayment schemes
	Withdrawing reimbursement (delisting)
	Prescription caps
	Coinsurance
	Educational approaches to increase generics use
	Regulating DTC advertising
***Policies Targeting Physicians***	Information and feedback to physicians on prescribing analysis and cost
	Budgetary restrictions—incentive systems
	Prescription guidelines
	INN prescribing (instead of brand names)
	Electronic prescription platform and information systems enabling audit and control
	Regulating drug promotion and the interaction between the pharmaceutical industry and physicians
***Policies Regulating the Pharmaceutical Industry***	Pharmacoeconomic evaluation/VBP/HTA
	Reimbursement—positive lists for reimbursed medicines
	Price regulation
	Budget programs and periodic price adjustments
	Tendering, centralized drug procurement programs and national drug price negotiation policies
	Special price agreements
	Budget cap and payback schemes (clawback)
	Escalating obligatory discounts to public hospitals and national healthcare funds (rebates) based on volume
	Generics pricing
	Prescription generic drugs price reduction
	Substitution of original drugs with generics and biosimilars
	Policies regulating mark-ups in the pharmaceutical supply and distribution chain
	Policies promoting drug price transparency
	OTC switch

DTC: Direct-to-Consumer; HTA: Health Technology Assessment, INN: International Non-proprietary Name (active ingredient name), OTC: Over-the-Counter; VBP: value-based pricing. Source(s): Authors’ work.

## Data Availability

Not applicable.

## References

[1] Mossialos E., Walley T., Mrazek M., Mossialos E., Mrazek M., Walley T. (2004). Regulating pharmaceuticals in Europe: An overview. Regulating Pharmaceuticals in Europe: Striving for Efficiency, Equity and Quality.

[2] Godman B., Malmström R.E., Diogene E., Gray A., Jayathissa S., Timoney A., Acurcio F., Alkan A., Brzezinska A., Bucsics A. (2015). Are new models needed to optimize the utilization of new medicines to sustain healthcare systems?. Expert Rev. Clin. Pharmacol..

[3] Freemantle N., Bloor K. (1996). Lessons from international experience in controlling pharmaceutical expenditure. I: Influencing patients. BMJ.

[4] Bloor K., Freemantle N. (1996). Lessons from international experience in controlling pharmaceutical expenditure. II: Influencing doctors. BMJ.

[5] Bloor K., Maynard A., Freemantle N. (1996). Lessons from international experience in controlling pharmaceutical expenditure. III: Regulating industry. BMJ.

[6] Berger M., Pock M., Reiss M., Röhrling G., Czypionka T. (2023). Exploring the effectiveness of demand-side retail pharmaceutical expenditure reforms: Cross-country evidence from weighted-average least squares estimation. Int. J. Health Econ. Manag..

[7] Guindon G.E., Fatima T., Garasia S., Khoee K. (2022). A systematic umbrella review of the association of prescription drug insurance and cost-sharing with drug use, health services use, and health. BMC Health Serv. Res..

[8] Lee I.H., Bloor K., Hewitt C., Maynard A. (2015). International experience in controlling pharmaceutical expenditure: Influencing patients and providers and regulating industry—A systematic review. J. Health Serv. Res. Policy.

[9] Lee I.H., Bloor K., Hewitt C., Maynard A. (2012). The effects of new pricing and copayment schemes for pharmaceuticals in South Korea. Health Policy.

[10] Soumerai S.B., Ross-Degnan D., Gortmaker S., Avorn J. (1990). Withdrawing payment for nonscientific drug therapy: Intended and unexpected effects of a large-scale natural experiment. JAMA.

[11] Theodorou M., Tsiantou V., Pavlakis A., Maniadakis N., Fragoulakis V., Pavi E., Kyriopoulos J. (2009). Factors influencing prescribing behaviour of physicians in Greece and Cyprus: Results from a questionnaire based survey. BMC Health Serv. Res..

[12] Polyzos N., Kastanioti C., Zilidis C., Mavridoglou G., Karakolias S., Litsa P., Menegakis V., Kani C. (2016). Greek national e-prescribing system: Preliminary results of a tool for rationalizing pharmaceutical use and cost. Glob. J. Health Sci..

[13] Kontodimopoulos N., Kastanioti C., Thireos E., Karanikas H., Polyzos N. (2013). The contribution of generic substitution to rationalizing pharmaceutical expenditure in Greek public hospitals under recent economic crisis. J. Pharm. Health Serv. Res..

[14] Rashidian A., Omidvari A.H., Vali Y., Sturm H., Oxman A.D. (2015). Pharmaceutical policies: Effects of financial incentives for prescribers. Cochrane Database Syst. Rev..

[15] Pelc A., Castan J.P. (1994). New developments in pricing and drug reimbursement in France. Pharmacoeconomics.

[16] Mason J., Freemantle N., Nazareth I., Eccles M., Haines A., Drummond M. (2001). When is it cost-effective to change the behavior of health professionals?. JAMA.

[17] Tisdale R.L., Ma I., Vail D., Bhattacharya J., Goldhaber-Fiebert J.D., Heidenreich P.A., Sandhu A.T. (2021). Availability of cost-effectiveness studies for drugs with high Medicare Part D expenditures. JAMA Νetw. Open.

[18] Kannarkat J.T., Good C.B., Parekh N. (2020). Value-based pharmaceutical contracts: Value for whom?. Value Health.

[19] Godman B., Bucsics A., Vella Bonanno P., Oortwijn W., Rothe C.C., Ferrario A., Bosselli S., Hill A., Martin A.P., Simoens S. (2018). Barriers for access to new medicines: Searching for the balance between rising costs and limited budgets. Front. Public Health.

[20] Emanuel E.J., Zhang C., Glickman A., Gudbranson E., DiMagno S.S.P., Urwin J.W. (2020). Drug reimbursement regulation in 6 peer countries. JAMA Intern. Med..

[21] Nguyen T.A., Knight R., Roughead E.E., Brooks G., Mant A. (2015). Policy options for pharmaceutical pricing and purchasing: Issues for low- and middle-income countries. Health Policy Plan..

[22] Verghese N.R., Barrenetxea J., Bhargava Y., Agrawal S., Finkelstein E.A. (2019). Government pharmaceutical pricing strategies in the Asia-Pacific region: An overview. J. Mark. Access Health Policy.

[23] Acosta A., Ciapponi A., Aaserud M., Vietto V., Austvoll-Dahlgren A., Kösters J.P., Vacca C., Machado M., Diaz Ayala D.H., Oxman A.D. (2014). Pharmaceutical policies: Effects of reference pricing, other pricing, and purchasing policies. Cochrane Database Syst. Rev..

[24] Ioannides-Demos L.L., Ibrahim J.E., McNeil J.J. (2002). Reference-based pricing schemes: Effect on pharmaceutical expenditure, resource utilisation and health outcomes. Pharmacoeconomics.

[25] Wushouer H., Luo Z., Guan X., Shi L. (2022). Influence of government price regulation on the price, volume and spending of antibiotics in China: A controlled interrupted time series study. Int. J. Health Policy Manag..

[26] Lin Y.S., Hsiao F.Y., Cheng S.H. (2024). Long-term effects of the global budget program and periodic price adjustment on antibacterial agents: A nationwide decomposition analysis between 2001 and 2016. Am. J. Infect. Control..

[27] Mills M., Kanavos P. (2020). Do pharmaceutical budgets deliver financial sustainability in healthcare? Evidence from Europe. Health Policy.

[28] Wang X., He X., Zhang P., Zhang M., Ma R., Dai R., Li X. (2023). The impact of the national volume-based procurement policy on the use of policy-related drugs in Nanjing: An interrupted time-series analysis. Int. J. Equity Health.

[29] Chen L., Yang Y., Luo M., Hu B., Yin S., Mao Z. (2020). The impacts of national centralized drug procurement policy on drug utilization and drug expenditures: The case of Shenzhen, China. Int. J. Environ. Res. Public Health.

[30] Wang J., Yang Y., Xu L., Shen Y., Wen X., Mao L., Wang Q., Cui D., Mao Z. (2022). Impact of ‘4+7’ volume-based drug procurement on the use of policy-related original and generic drugs: A natural experimental study in China. BMJ Open.

[31] Yuan J., Lu Z.K., Xiong X., Lee T.Y., Huang H., Jiang B. (2022). Impact of national volume-based procurement on the procurement volumes and spending for antiviral medications of Hepatitis B Virus. Front. Pharmacol..

[32] Cai L., Tao T., Li H., Zhang Z., Zhang L., Li X. (2022). Impact of the national drug price negotiation policy on the utilization, cost, and accessibility of anticancer medicines in China: A controlled interrupted time series study. J. Glob. Health..

[33] Main B., Csanadi M., Ozieranski P. (2022). Pricing strategies, executive committee power and negotiation leverage in New Zealand’s containment of public spending on pharmaceuticals. Health Econ. Policy Law.

[34] Dunlop W.C., Staufer A., Levy P., Edwards G.J. (2018). Innovative pharmaceutical pricing agreements in five European markets: A survey of stakeholder attitudes and experience. Health Policy.

[35] Mitkova Z., Dimitrova M., Doneva M., Tachkov K., Kamusheva M., Marinov L., Gerasimov N., Tcharaktchiev D., Petrova G. (2022). Budget cap and pay-back model to control spending on medicines: A case study of Bulgaria. Front. Public Health.

[36] Letsios A.N., Mavridoglou G., Ladopoulou D., Tsourdini D., Dedes N., Polyzos N.M. (2023). Exploring the impact of clawback on pharmaceutical expenditure: A case study of public hospitals in Greece. Int. J. Health Plan Manag..

[37] Memedovich K.A., Manns B., Beall R., Hollis A., Clement F. (2019). The impact of pharmaceutical rebates on patients’ drug expenditures. CMAJ.

[38] Wouters O.J., Kanavos P.G. (2017). A comparison of generic drug prices in seven European countries: A methodological analysis. BMC Health Serv. Res..

[39] Li Y. (2023). Generic price regulation and drug expenditures: Evidence from Canada. Value Health.

[40] Kim W., Koo H., Lee H.J., Han E. (2022). The effects of cost containment and price policies on pharmaceutical expenditure in South Korea. Int. J. Health Policy Manag..

[41] Mishuk A.U., Fasina I., Qian J. (2020). Impact of U.S. federal and state generic drug policies on drug use, spending, and patient outcomes: A systematic review. Res. Soc. Adm. Pharm..

[42] Chai Q., Wen H., Lang Y., Zhang L., Song Y., Liu X. (2022). Budget impact analysis of the introduction of a trastuzumab biosimilar for HER2-positive breast cancer in China. Clin. Drug Investig..

[43] Gomes T., McCormack D., Kitchen S.A., Paterson J.M., Mamdani M.M., Proulx L., Bayliss L., Tadrous M. (2021). Projected impact of biosimilar substitution policies on drug use and costs in Ontario, Canada: A cross-sectional time series analysis. CMAJ Open.

[44] Joosse I.R., Tordrup D., Glanville J., Mantel-Teeuwisse A.K., van den Ham H.A. (2023). A systematic review of policies regulating or removing mark-ups in the pharmaceutical supply and distribution chain. Health Policy.

[45] Joosse I.R., Tordrup D., Glanville J., Kotas E., Mantel-Teeuwisse A.K., van den Ham H.A. (2023). Evidence on the effectiveness of policies promoting price transparency—A systematic review. Health Policy.

[46] Gibson T.B., Mark T.L., McGuigan K.A., Axelsen K., Wang S. (2006). The effects of prescription drug copayments on statin adherence. Am. J. Manag. Care.

[47] Lexchin J., Grootendorst P. (2004). Effects of prescription drug user fees on drug and health services use and on health status in vulnerable populations: A systematic review of the evidence. Int. J. Health Serv..

[48] O’Malley A.J., Frank R.G., Kaddis A., Rothenberg B.M., McNeil B.J. (2006). Impact of alternative interventions on changes in generic dispensing rates. Health Serv. Res..

[49] Martens J.D., Werkhoven M.J., Severens J.L., Winkens R.A. (2007). Effects of a behaviour independent financial incentive on prescribing behaviour of general practitioners. J. Eval. Clin. Pract..

[50] Chen C., Dong W., Shen J.J., Cochran C., Wang Y., Hao M. (2014). Is the prescribing behavior of Chinese physicians driven by financial incentives?. Soc. Sci. Med..

